# Confidence Interval Estimation for Cutting Tool Wear Prediction in Turning Using Bootstrap-Based Artificial Neural Networks

**DOI:** 10.3390/s24113432

**Published:** 2024-05-26

**Authors:** Lorenzo Colantonio, Lucas Equeter, Pierre Dehombreux, François Ducobu

**Affiliations:** Machine Design and Production Engineering Lab, Research Institute for Science and Material Engineering, Research Institute for the Science and Management of Risks, University of Mons, 7000 Mons, Belgium; lucas.equeter@umons.ac.be (L.E.); pierre.dehombreux@umons.ac.be (P.D.); francois.ducobu@umons.ac.be (F.D.)

**Keywords:** cutting tool, artificial intelligence, reliability, monitoring, degradation

## Abstract

The degradation of the cutting tool and its optimal replacement is a major problem in machining given the variability in this degradation even under constant cutting conditions. Therefore, monitoring the degradation of cutting tools is an important part of the process in order to replace the tool at the optimal time and thus reduce operating costs. In this paper, a cutting tool degradation monitoring technique is proposed using bootstrap-based artificial neural networks. Different indicators from the turning operation are used as input to the approach: the RMS value of the cutting force and torque, the machining duration, and the total machined length. They are used by the approach to estimate the size of the flank wear (VB). Different neural networks are tested but the best results are achieved with an architecture containing two hidden layers: the first one containing six neurons with a Tanh activation function and the second one containing six neurons with an ReLu activation function. The novelty of the approach makes it possible, by using the bootstrap approach, to determine a confidence interval around the prediction. The results show that the networks are able to accurately track the degradation and detect the end of life of the cutting tools in a timely manner, but also that the confidence interval allows an estimate of the possible variation of the prediction to be made, thus helping in the decision for optimal tool replacement policies.

## 1. Introduction

In the manufacturing industry, turning plays a major role in the production of parts. This machining operation inevitably induces tool wear which comes from various degradation mechanisms occurring simultaneously at the interface between tool and workpiece [1,2]. This decrease in tool performance implies that some machining specifications cannot always be met, resulting in the production of poor quality parts. Under nominal cutting conditions, wear is mainly located on the flank face of the tool, and its evolution is variable, even for stable cutting parameters. It is therefore necessary to replace the tool regularly, in order to optimize production and reduce costs [3]. It is estimated that tool costs can account for up to 12% of the production costs of a part [4]. Thus, to ensure that tools operate under nominal conditions and to minimize tool costs, the monitoring of cutting tools has become an important research topic [5]. Multiple applications of monitoring have been proposed in the literature to optimize cutting parameters [6], estimate workpiece surface roughness [7], or estimate tool life [8]. The latter is one of the most popular research topics, focusing on estimating tool wear using the VB indicator (width of the flank wear). Multiple types of models attempt to perform this task, initially via empirical laws [8], then by statistical models [9], and, more recently, through artificial intelligence (AI) models with instrumented machining operations [10]. AI performs particularly well compared to other methods, and almost always outperforms them. Nonetheless, special attention to noise and outliers in the database is needed to train the AI properly [11].

There exist two types of tool condition monitoring: direct and indirect [12]. The direct monitoring method involves measuring tool wear directly on the insert by stopping the machining process to make the measurement. There exists several methods to measure the wear on the tool: laser profilometry, stereo vision, micro or macrophotography, fringe projection profilometrty, etc. [13]. Multiple image processing adapted to each measurement method exists, but due to the complexity of the task, the use of some deep learning approaches becomes more and more common [14]. In practice, the direct measurement approach has its limitations. Due to the continuous nature of the cutting process, the machining needs to be stopped in order to make measurement, thus reducing the productivity of the machine. In addition, special attention to illumination condition and reflectivity of the material is necessary in many cases which makes industrial applications more complex due to the use of cutting fluids for example [15].

Indirect methods, on the other hand, rely on signals recovered during machining to determine the condition of the tool. These signals, unlike those collected by direct monitoring, are measured during the machining operation. Those are, as examples, the cutting forces, the vibrations, etc. A complete description of all the measurable signals in indirect monitoring during machining is extensively reviewed in [16]. To monitor cutting tool wear, AI methods can be used to perform classification task. Classification methods consist of using AI to estimate the state of the tool, generally divided into different categories representing the different stages of the tool life (new to worn tool). A comparison of the performance of three different AI approaches—artificial neural networks, support vector classifier, and K-nearest neighbor—to classify tool state using cutting force is proposed in [17]; the authors showed that an ANN can outperform other AI methods. Other studies, this time using accelerometers, also propose classifying the state of the tool, but they use unsupervised learning methods such as self-organizing maps on imbalanced data and compared them with more classical supervised ones. It is shown that this type of approach can detect the different regimes of tools with only one sensor [18]. Another approach, based on the knowledge that a worn tool will operate at higher temperature conditions, uses thermographic images to successfully classify the state of a tool [19]. Based on a similar observation, another approach uses the color of chips to classify the condition of a tool [20]. Although all these methods are capable of correctly classifying the state of a tool, in some applications, classification is not sufficient. By its very nature, classification also fails to highlight intermediate cases between two classes. For example, a tool could correctly be classified as still being usable while being only a few microns away from being unusable. To avoid this situation, some researchers try to estimate the value of VB directly, this time by regression. As with classification, several methods exist, each using different AI and cutting signals. In [21], a feature selection technique is developed to identify the best set of features from acoustic emission signals. This technique is then applied to multiple AI approaches such as artificial neural networks, support vector machine, etc. Other authors used the cutting forces with an adaptive neuro-fuzzy inference system to track the degradation of tool wear [10]. Some approaches also focus on materials with a reputation of being hard to cut, such as Inconel 718. This material is used in [22], where the cutting forces, acoustic emissions, and vibrations are processed with wavelet packet transform to extract relevant features. These features are then selected through a Pearson correlation analysis and the most relevant ones are used to feed a neural network. The latter is used to estimate the wear of the tool. Several reviews of the literature summarize all the possible approaches and features processing methods [5,23]. From these reviews, it can be observed that the most common approach, and one capable of obtaining good results, is the use of neural networks. This approach is by far the one which is employed the most in the literature for its ability to model highly nonlinear relationships and its ability to adapt to different signals and cutting conditions more efficiently than the others. On average, the neural networks used are fairly shallow, with only a few neurons in each layer. Although the deep learning method can be used, it is sometimes preferable to select useful features using expert knowledge, which is why deep convolutional neural networks are sometimes not selected for this monitoring task. Some authors have also noted that using too many signals can have a detrimental effect on the performance of the methods [24]. Although articles on the subject monitor wear evolution with varying degrees of accuracy, the confidence interval around tool wear estimates is never considered. Furthermore, the natural variability in tool wear in machining processes [25] leads to a situation in which different tools—even though they are used in identical cutting conditions—exhibit highly different wear evolution. Any model that produces a single value estimate places the practitioner in a difficult position when assessing how accurate the prediction is [26]. In practice, a confidence interval or distribution is required in order to allow an estimation of the reliability of the prediction [27]. This, in turn, allows one to estimate how likely it is that a tool would fail to meet quality requirements over the next machining operation; hence, one can estimate whether a tool replacement should be performed now or later.

In this article, we propose that the degradation of cutting tools should be monitored by using cutting forces and neural networks. The novelty of the approach presented lies in the establishment of a confidence interval around the monitoring of the wear. In order to estimate the confidence interval around the estimate, it is proposed that a method called bootstrap [28,29] is used. The bootstrap method consists of using the inference of multiple neural networks in parallel to create an interval of confidence around the estimation of wear. As highlighted above, the literature on monitoring the degradation of cutting tools never considers a confidence interval. Yet, this parameter is extremely important in any decision-support method. The aim of this article is therefore to test the establishment of such a confidence interval estimation method. The approach is based on data recovered from turning tests. The models are trained on constant machining conditions and tested on trajectories with variations in cutting speed. The aim is to test the ability of the approach to monitor tool degradation in previously unseen cutting variations and to observe the behavior of the confidence interval in these situations.

## 2. Methodology

**Figure 1:**
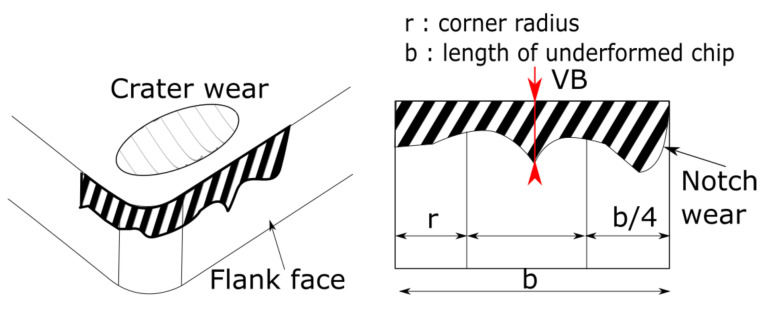
Tool degradation and VB.

**Figure 2:**
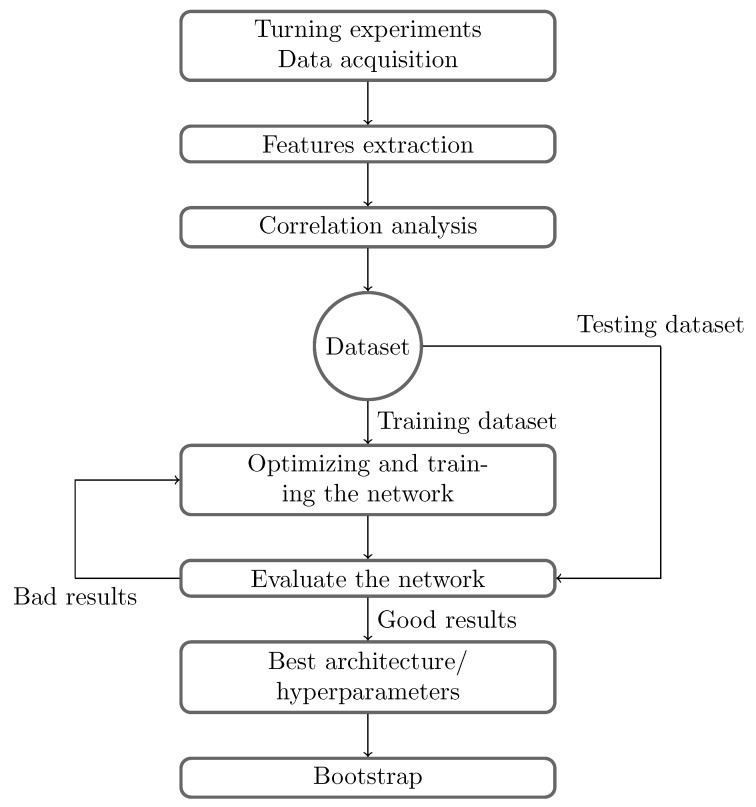
Methodology flow chart.

This paper proposes an approach to designing a bootstrap-based neural network able to monitor tool degradation in real time with a confidence interval estimation. As it is commonly used, the degradation of a tool is characterized by the evolution of VB, which is defined as the value of the width of the flank wear edge in ISO 3685 [30] (Figure 1). The flow chart (Figure 2) illustrates the whole approach. The flowchart is realized as follows:

- A database containing mainly cutting forces from turning tests is used.
- The database, containing the raw force data, needs to be processed. As commonly realized in AI, these temporal force signals are preprocessed to obtain indicators, either statistical (root mean squared, skewness, etc.) or frequency (power spectral density).
- These indicators are not all correlated with the wear of the cutting tool; a selection of the most correlated indicator is therefore necessary to avoid using irrelevant information to train the network. Therefore, to find the best input for the networks, a correlation analysis is performed to establish the correlation between the tool wear and the computed indicator. An indicator with a high correlation to the tool wear is then used as an input for the the neural network. These inputs are standardized.
- The network is optimized to find the best architecture and combination of hyperparameters in order to achieve the best results. Once the best architecture is identified, the bootstrap method is applied to estimate the confidence interval around the prediction.
- The results are tested on previously unseen cutting speed variations in order to assess the capability of the network to generalize the results.

The following part of the text describes each step of this workflow.

### 2.1. Experimental Setup

**Figure 3:**
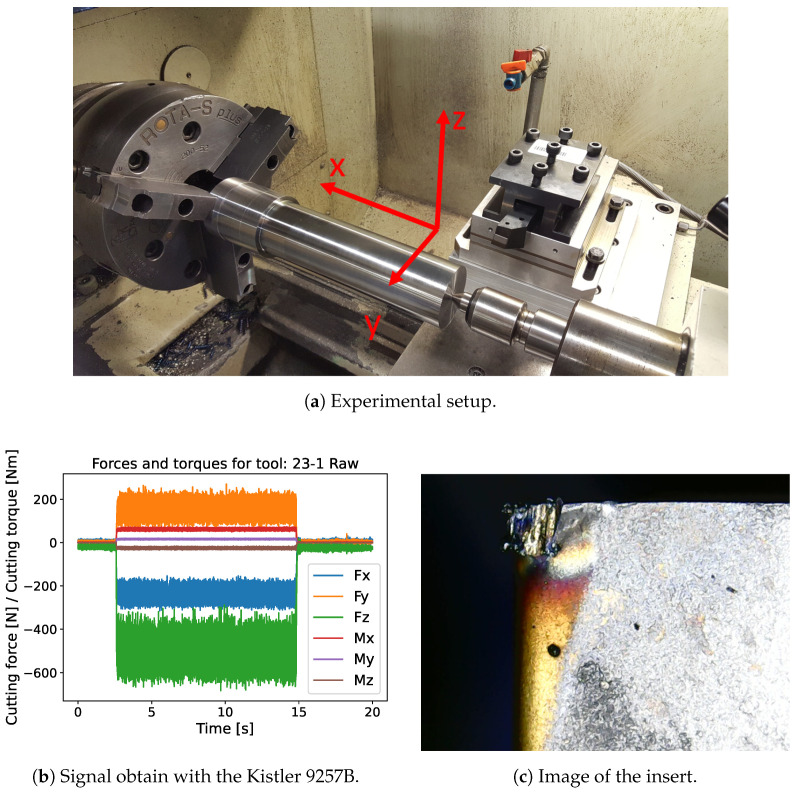
Illustration of various signals from the dataset.

The database used in this paper is derived from experimental turning tests of C45 steel bars that received a heat treatment at 850 °C during 8 h (annealing) to achieve a constant hardness through the section of 179 HV30. The CNC lathe is the Weiler E35 (Figure 3a), on which the insert and tool holder are chosen according to SECO TOOLS recommendations [31]. The insert is the CNMG120404-M3 TP40 and the tool holder is the DCLN L/R 2020K12-M. The insert is of the lowest grade to reduce its lifespan, thus reducing the wasting of material during the tests. The database consists of the degradation of 30 tools operating at different cutting speeds. Table 1 shows the cutting conditions for each tool.

Different types of signals are recorded during machining, as shown in Table 2. The main information is the cutting force and torque, which are measured by a Kistler 9257B force sensor. These forces and torques are measured in all spatial directions during a 20 s signal sampled at 10 kHz. In the reference frame, $F_{x}$ is the feed force, $F_{y}$ is the radial force, and $F_{z}$ is the cutting force. At regular intervals (2.8 min), the machining is stopped in order to measure tool degradation. This is realized by direct measurements on the tool through a Byameyee EU-1000X 3 digital portable microscope (Figure 3c). From the images of the tool insert, the degradation was measured based on the ISO 3685 standard [30]. The experimental setup yielded 192 measurements points spread over the lifetime of 30 tools; on average, 6.4 measurements were made per tool. This number of tools is higher than what is generally reported in the literature. Indeed, about ten tools are generally used to test the methods, as is the case in [22,32,33]. In the presented database, more tools are considered showing dispersion in degradation even under identical cutting conditions. To ensure that our database contains sufficient data and that adding data would not improve the results, data augmentations were attempted. However, the quality of the results was not improved by data augmentation. Therefore, the results on the non-augmented database are presented in this article.

### 2.2. Features Extraction

The temporal signal of the cutting forces is not directly usable with the neural networks used below, so it is necessary to extract relevant features from these signals. There are several techniques for feature extraction, the most common being statistical and frequency analysis of the signal. In the following, 5 statistical and 2 frequency features are computed for each cutting forces and torques ($F_{X}$, $F_{Y}$, $F_{Z}$, $M_{X}$, $M_{Y}$, and $M_{Z}$).

**Figure 4:**
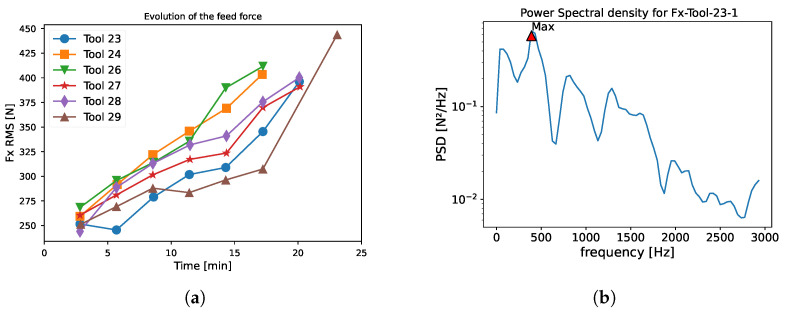
Example of features extracted. (**a**) RMS feed force for multiple tools. (**b**) Power spectral density on the feed force, estimated with the Welch’s method.

The different statistical feature extraction techniques have been chosen based on the literature on the subject [23]: root mean squared (RMS), mean, variance, kurtosis, and skewness. If the temporal signals from the sensors are represented as $t_{1}$,…,$t_{n}$, then the statistical features can be computed as presented in Table 3; the central moment ($m_{i}$) is defined in Equation (1). Figure 4a shows the evolution of the RMS value of the feeding force ($F_{X}$) for different tools.

(1) $$m_{i}=\frac{1}{N}\sum_{i=1}^{N}{\left(t_{i}-\bar{t}\right)}^{i}$$

In the frequency domain, the Welch’s method [34] is used to extract the power spectral density (PSD) on each signal ($F_{X}$, $F_{Y}$, $F_{Z}$, $M_{X}$, $M_{Y}$, and $M_{Z}$). Figure 4b shows the PSD under 3 kHz computed with the Welch’s method on the feed force ($F_{X}$) for the first measurement point of tool 23. The amplitude and the associated frequency of the highest peak are used as indicators.

Other features can also be computed, such as the total length machined by the tool; this can be computed as (Equation (2)):

(2) $$\mathrm{total}\;\mathrm{machined}\;\mathrm{length}=\int V_{c}\left(t\right)dt$$

### 2.3. Correlation Analysis and Features Selection

Not all of the features extracted with the previous methods are identically correlated with cutting tool wear. In order to identify those most related with it, a correlation analysis is performed. Many features selection methods exist; in accordance with the approach commonly used in the literature on the topic, it is proposed that we use a correlation analysis [22]. A correlation analysis is a statistical method used to evaluate the relationship between two variables. A correlation coefficient equal to 1 implies perfect correlation between the two values. Multiple types of correlation analysis exist, in this article the Spearman’s correlation analysis is used as it is the most adapted to the size and non-normality of the dataset [35,36].

Table 4 shows the 5 selected features and their correlation with tool wear. In the dataset, these features are the most correlated with the wear. The quantity of 5 inputs is chosen after tests where the addition of more inputs did not improve the results. In order to keep the network as small as possible, the smallest number of inputs is chosen. Of these 5 signals, 3 are related to the cutting forces ($M_{z}$ RMS, $F_{x}$ RMS, and $F_{z}$ RMS) and 2 are independent of the tool condition (machining duration and total machined length) but provide information about the cutting condition. To be used by neural networks, input data must be standardized. Therefore, all the results presented in the following will use standardized data.

The database is composed of cutting force signals, inhibiting a comparison of the correlation of this signal with other signals, such as vibrations, which are regularly used in this type of study [23,37]. Nevertheless, a strong correlation between cutting forces and tool degradation state is observed; this is also observed in the literature [38,39]. The machined length and machining time are also related to the tool wear, as it is the image of the amount of material already machined and is therefore a good indicator for the tool condition.

In the database, there are only the cutting speed variations for the cutting condition. It should be noted that, for industrial production applications, the cutting condition must be used in inputs of the neural network to identify the working regime of the tool. With the current database, there are no significant variations in cutting conditions that would drastically change the degradation trajectories, but this still enables us to observe trends in the proposed approach.

### 2.4. Training and Testing Dataset

The objective of a neural network is to learn from a training dataset in order to be able to generalize its learning to previously unseen data. A common risk in this process is the risk of overfitting the neural network to the training dataset. This overfitting takes place when the network memorizes the training data and it is often accompanied by low accuracy on the network to new data. To avoid overfitting, the database should be split and the learning of the network monitored [40]. A common approach is to divide the dataset into three parts: a training set, a validation set, and a test set. The training and validation sets are used during the training of the network to monitor the learning, while the test set is used to test the ability of the network to generalize its results. In the following, the testing set represents 20% of the whole dataset. The remaining 80% are split as follows: 75% for the training set and 25% for the validation set. These values are common practice in artificial intelligence; also, all data are normalized to ensure faster convergence and better results.

**Figure 5:**
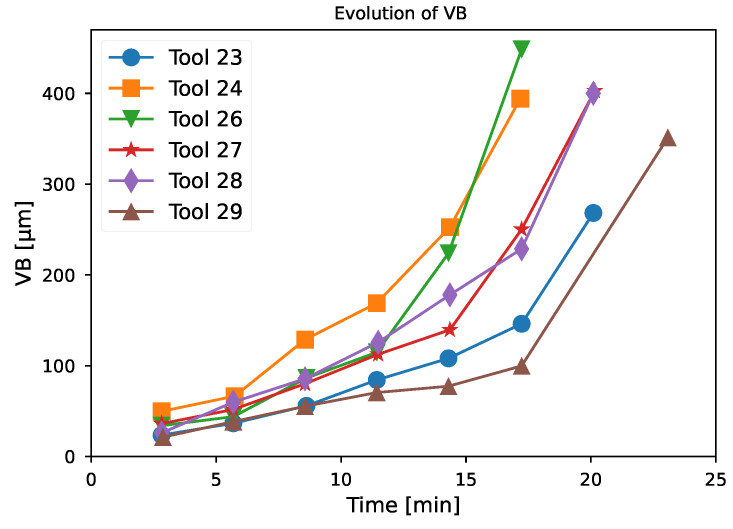
Degradation trajectory for tools 23, 24, 26, 27, 28, and 29.

To ensure that the neural network is able to generalize its results to new cutting conditions, the test set consists of the tool number: 23, 24, 26, 27, 28, and 29. These tools were selected as the testing dataset from those who saw variable cutting speed between two measurements (tools 20 to 30). Figure 5 shows the degradation of the selected tool. Each tool has a different degradation trajectory; some, such as tool 23, have an almost linear degradation, whereas tool 26 has a brutal degradation after 11 min of machining. These particularities in their degradation trajectories make them an excellent testing set.

It should be noted that the measurement of VB has some measurement errors that are not possible to reliably estimate and thus are not taken into account in the figures. The ISO 3685 standard specifies that VB should be measured using the average of the flank wear. However, the standard does not specify a minimum number of measurements for estimating this average. This problem is usually not discussed in the literature but can impact the value of VB. Our measurement method consists of identifying the wear area using image processing software and then calculating the value of VB along the entire length of the area. This allows the estimation of the mean value of VB to be made as accurately as possible. This method provides an estimate of VB that is better than the average of a few points. It also averages out any errors that may have been made when selecting pixels at the border of the wear area in the image. In this context, it can be estimated that this measurement error is low and thus does not modify the results presented in this article.

### 2.5. Hardware and Software for Neural Network

The software resources and their applications, used to create the neural network, are listed in Table 5. For the training, a CPU Intel I7-9750h (6 cores and 12 threads at 2.6 GHz–4.5 GHz) is used.

The different hyperparameters defining the network and its learning have been defined by trial and error and by reviewing the literature on the subject. The networks are optimized with the Adam optimizer, which minimizes the mean square error between the actual and predicted values of VB. The learning rate is first set to 0.001 and the learning is monitored so that if the network stops learning, the learning rate is reduced to refine the results. This is accomplished with a Keras callback called “ReduceLROnPlateau” [41]. The batch size is set to 5 and the maximum number of epochs is set to 1000. In order to avoid training the network too long after convergence and therefore wasting computing time, we use a keras function called “EarlyStopping” [41]. This “EarlyStopping” is triggered if the network has not improved its results after 75 epochs. During these 75 epochs of non-improvement, we try to refine the results by changing the learning rate parameters. This is performed using the Keras “ReduceLrOnPlateau” function [41]. If, despite changing the learning rate and after 75 epochs, the network is no longer able to improve its results, the training is stopped. This approach allows us to limit the risk of overfitting and also ensure that the selected model had the opportunity to converge before stopping the learning process.

### 2.6. Determination of the Best Network Architecture

**Figure 6:**
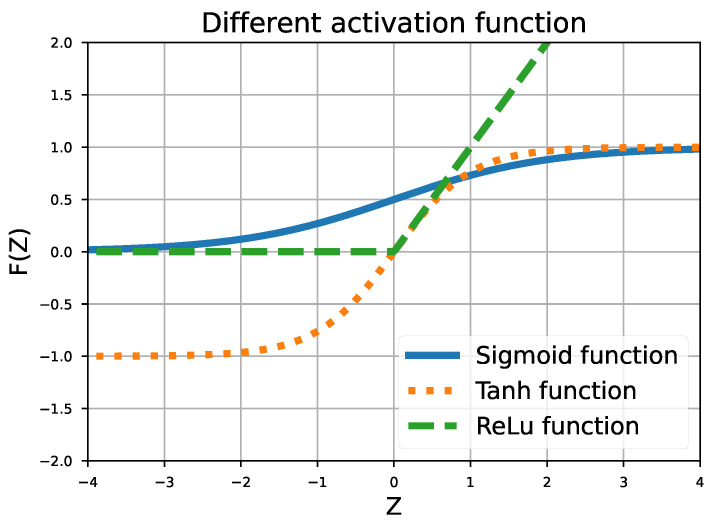
Sigmoid, Tanh, and ReLu activation functions.

The training of neural networks depends mainly on the choice of hyperparameters and the architecture of the network. The literature on the subject generally uses neural networks with a shallow architecture: 1–2 hidden layers with a few neurons inside [23]. Therefore, similar combinations of architectures, containing between 1 and 4 hidden layers each with between 5 and 15 neurons, were tested. To ensure that more complex models would not be more appropriate, we also tested models with up to 40 hidden layers. These models did not perform better than the ones presented in the following. The different architectures are tested on the same test dataset and the mean square error is used as an indicator to compare their performances. Different combinations of activation functions are tested. Figure 6 shows the most common activation functions used: Sigmoid, Tanh, and ReLu.

Table 6 compares the relative performance of different architectures on the test dataset (Section 2.4). Several indicators are presented: mean squared error (MSE), the coefficient of determination (R^2^ score), and mean absolute percentage error (MAPE). These indicators are defined as follows:

- The MSE is a measure of how close a fitted line is to data points. For each point, it calculates the square difference between the observed and predicted values and then averages these values. The lower the MSE, the better the prediction performance. The MSE is defined as (Equation (3)):
  (3) $$\mathrm{MSE}=\frac{1}{n}\sum_{i=1}^{n}{\left(Y_{i}-{\hat{Y}}_{i}\right)}^{2}$$
  where:
  – *n* is the total number of observations;
  – $Y_{i}$ is the actual value of the $i^{\mathrm{th}}$ observation;
  – ${\hat{Y}}_{i}$ is the predicted value of the $i^{\mathrm{th}}$ observation.
- The R^2^ score is a statistical measure that represents how well the predicted value fit to the real one. A value of 1 indicates a perfect fit, while a value of 0 shows no fit. The R^2^ score is defined as (Equation (4):
  (4) $$R^{2}=1-\frac{\sum_{i=1}^{n}{\left(Y_{i}-{\hat{Y}}_{i}\right)}^{2}}{\sum_{i=1}^{n}{\left(Y_{i}-\bar{Y}\right)}^{2}}=1-\frac{SS_{res}}{SS_{tot}}$$
  where:
  – $\bar{Y}$ is the mean of the actual values;
  – $SS_{res}$ is the sum of squares of the residual errors;
  – $SS_{tot}$ is the total sum of squares.
- The MAPE is a statistical measure used to determine the accuracy of a prediction. It calculates the average of the absolute percentage differences between the actual and predicted values. Lower MAPE values indicate better accuracy. A MAPE value of 0 indicates perfect predictions. It is expressed as a percentage, and is defined by the formula (Equation (5)):
  (5) $$\mathrm{MAPE}=\frac{100\%}{n}\sum_{i=1}^{n}\left|\frac{Y_{i}-{\hat{Y}}_{i}}{Y_{i}}\right|$$

These scores are calculated on the average value of the monitoring on the test trajectories presented in Section 2.4. The wear range of these trajectories extends from 0 to 450 μm. The notation [5, 6, 6, 1] is read as there are 5 inputs, 2 hidden layers each containing 6 neurons, and 1 output. Other, deeper, architectures have been tested and are not included in the table as their performances are lower. Several observations can be made about Table 6.

- All architectures containing only “ReLu” layers converge to similar performance, as shown by their comparable performance indicators. The mean R^2^ score stands around 88%, while the mean MAPE is around 23%. The architecture seems to have minimal impact on the results obtained. Consequently, it is recommended to use less complex network architectures, as more complex ones do not offer significant advantages for databases such as the one presented in this article.
- The architectures with only Tanh or Sigmoid activation function fail to obtain good results in only 1000 epochs. This is largely attributed to the data scale. Specifically, Tanh and Sigmoid functions can only output values ranging from −1 to 1 and 0 to 1, respectively. Given that the VB values are expressed in microns (ranging from 0 to 450 μm), a saturation effect is observed. It is important to note that, even when the VB values are scaled to better suit these architectures, the results do not show any significant improvement over those discussed in the following.
- The optimal architecture is composed of two hidden layers, each with six neurons. The first layer utilizes a Tanh activation function, while the second uses an ReLu activation function. Compared with approaches using only ReLu, this architecture improves the R^2^ score by an average of 5% and MAPE by 8%.

**Figure 7:**
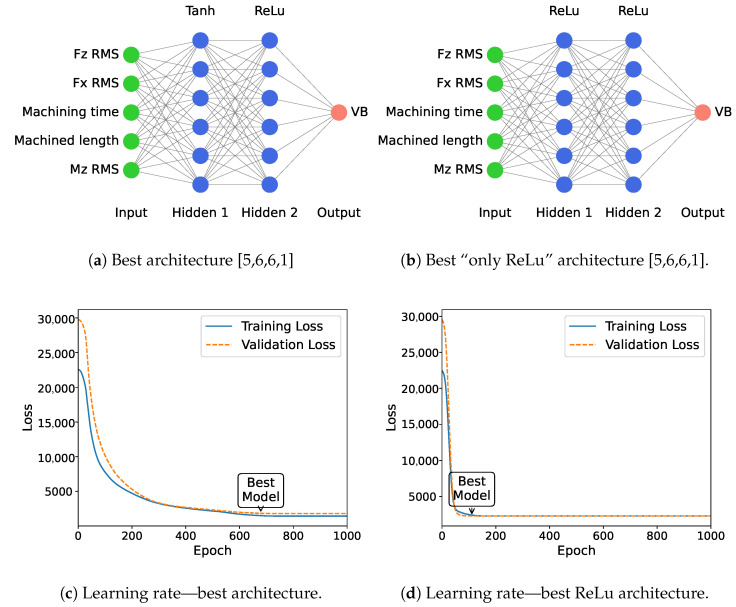
Learning comparison between the best identified model and an uniform network—note that the “EarlyStopping” is disabled to allow us to have 1000 epochs and not be stopped after convergence.

It should be noted that the network with a Tanh layer (Figure 7a) takes longer to train than a network with only ReLu layers (Figure 7b). This is observed in Figure 7c,d, where the best model takes 4 times longer to converge but achieves better results on the testing set. These figures also show that there is no overfitting, as the training and validation losses have a similar evolution.

### 2.7. Confidence Interval Evaluation

Although, in theory, neural networks have an excellent ability to approximate nonlinear behavior, they only provide a point estimate of the tool state. Thus, the prediction is less reliable if the data are sparse or affected by probabilistic events. To interpret the results correctly, the uncertainty of the network on the prediction must be taken into account [28]. In this paper, a bootstrap method is chosen to estimate the confidence interval of the neural network. This method has already been used in the literature but for different applications [29,42]. It involves training a set of neural networks in parallel and querying all the networks to estimate a confidence interval. The collective decision of the networks is less likely to be in error than any individual neural network. As each network does not converge in the same way due to the random initialization of the network and the training process, it will be shown that the predictions follow a normal distribution. Thus, if *J* is the total number of networks and $\hat{Y_{i}}$ the $i^{\mathrm{th}}$ estimation of the tool wear, then the average regressed value $\bar{Y_{P}}$ is (Equation (6)):

(6) $$\bar{Y_{P}}=\frac{1}{J}\sum_{i=1}^{J}\hat{Y_{i}}$$

The normality of the results imply that, by knowing the mean and standard deviation, it is possible to create a 95% confidence interval (CI) around the prediction (Equation (7)):

(7) $$CI=\bar{Y_{P}}\pm 1.96\sigma$$

**Figure 8:**
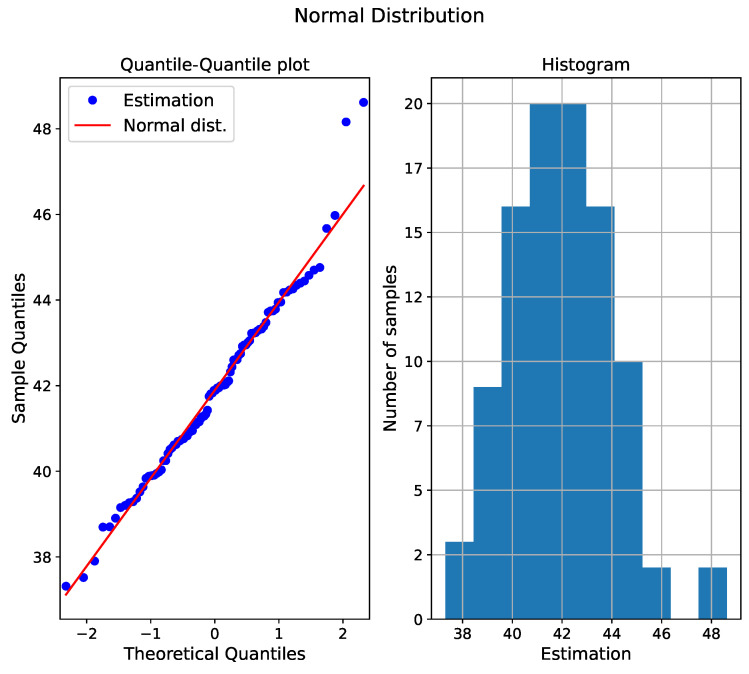
Quantile–quantile plot and histogram of the estimation of the neural networks.

The normality assumption can visually be proved by a quantile–quantile plot (Q-Q plot) [43]. Figure 8 shows the Q-Q plot as well as the histogram of the wear estimate for a cutting tool degradation with the network architecture previously presented. In order to have a significant number of points and thus a sufficient statistical distribution, this figure is made with 100 estimates from 100 networks trained with the bootstrap method. On the quantile–quantile graph, the red line represents the theoretical normal distribution; the blue points represent the different neural network estimates. If the distribution was perfectly normal, then all points would lie on the red line. In Figure 8, outliers are present at both ends, but it is observed that the majority of the points approximate the theoretical normal distribution. This confirms that the hypothesis of a normal distribution is respected. The second plot in Figure 8 is a histogram that allows us to visually represent the distribution; a bell-shaped curve is observable, confirming the normal distribution. This figure only depicts one estimate, but in general, a predominantly normal distribution is observed for all estimates.

In the following approach, this bootstrap method will be used to identify the confidence interval on the tool wear estimate. A total of 100 neural networks will be trained in parallel to create the confidence interval estimate. This number was identified by trial and error; the number of 100 is a compromise between the computational time for training and the behavior of the confidence interval. Beyond 100 networks, the confidence interval remains the same.

Compared to a classical neural network approach, this bootstrap approach requires more computing resources, especially during training. As mentioned above, the networks are relatively small, so they individually do not require high computing power. Training a neural network on a Intel I7-9750h (6 cores and 12 threads at 2.6 GHz to 4.5 GHz) takes less than a minute on a single core; training 100 networks in parallel takes around 10 min of computing time. Once the network is trained, integrating the 100 networks takes less than a second, which is not critical for real-time monitoring, even with low computing resources. It should be noted that the number of networks is overestimated; similar results could be possible with fewer networks, but for this article, it was decided that we would use the largest possible statistical distribution to demonstrate the approach.

## 3. Results

**Figure 9:**
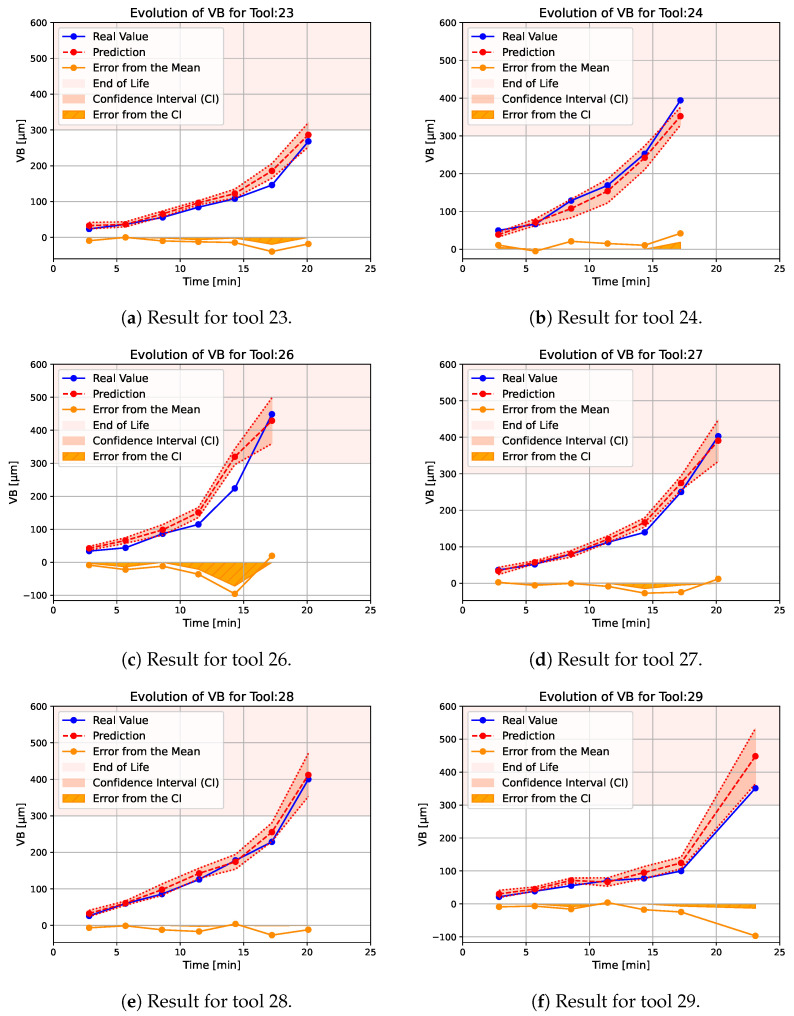
Estimated tool wear on the testing dataset.

Figure 9a–f show the results of the tool wear estimation with 100 neural networks with the architecture presented previously. The pink “End Of Life” area corresponds to an end of life criterion of VB equal to 300 μm as recommended in ISO 3685 [30]. The blue line corresponds to the actual measured degradation. The red line corresponds to the estimate by the neural network, while the pink area corresponds to the 95% confidence interval around the estimate. The orange area is the error between the confidence and the measured value, whereas the orange line is the error between the mean estimate and the measured value. The results for the different tools are as follows:

- For tool 23 (Figure 9a), the estimate is consistently slightly higher than the actual observed wear, but the confidence interval almost consistently matches the real value. A more significant overestimation is observed around 17 min that is around 40 μm, but the confidence interval is larger than before and the error on this interval is smaller. For this tool, the end of life has not been reached.
- Compared to the rest of the test database, tool 24 has a higher initial degradation rate. The network estimate has a slightly larger confidence interval over this part of the tool’s life, which is a unique case—generally, the confidence interval increases with time (Figure 9b). Apart from this sudden change in tool wear, the estimator is able to track the degradation correctly, but tends to slightly underestimate the value of the degradation. This is a unique case in this test dataset. For this tool, the observed end of life is reached less than 1 min before the predicted end of life.
- Tool 26 is the tool with the fastest end-of-life degradation (Figure 9c). At the beginning of the tool’s life, the network is able to track the degradation correctly with a relatively low confidence interval; then, due to the sudden change in the wear rate, the estimator strongly overestimates the tool’s degradation. In this case, it even indicates that the tool has reached the end of its life because the prediction around 15 min is higher than 300 μm. Then, the confidence interval increases strongly and the estimate is closer to reality. For this tool, the end of life is reached around 2 min after the predicted end of life.
- The degradation of tools 27 and 28 are fairly similar (Figure 9d,e), there is simply a slightly earlier change in the degradation rate for tool 27 than for tool 28. For both tools, the neural network correctly tracks the degradation and the confidence interval increases slightly at the end of the tool’s life. For these tools, the end of life is reached almost at the same time as the predicted ones.
- Tool 29 has a lower degradation rate than the others and a longer tool life (Figure 9f). The estimator is able to follow the degradation of the tool correctly, but its end of life is overestimated and the confidence interval is significantly larger than for the other tools. For this tool, the end of life is reached 2 min after the predicted end of life.

The results on particular degradation trajectories with either variable degradation rates or variable duration and with variation of the cutting speed show that the approach is able to follow the tool degradation correctly. On average, it is observed that the crossing of the end-of-life zone (300 μm of wear) is crossed in the same minute as the measured one. In the worst cases, the error produced on the estimation of the tool is not very significant and corresponds to an error of a few microns. There are, nevertheless, two erroneous points, one for tool 26 and the second for tool 29. The origin of this error is not clearly identifiable and all the other approaches tested made the same error at these points. However, we can note that, following these errors, the interval of confidence has increased, showing the interest of this approach. This interval is particularly useful when the tool sees sudden variations in wear and a single estimate would not allow an assessment of the network’s confidence. As the database does not contain significant variation in cutting conditions, the size of the confidence intervals is consistently small. This is interesting, and unexpected, because too-large confidence intervals would not allow us to clearly estimate the state of wear of the tool. Too-large confidence intervals in this article would have shown that the method would not be applicable in more complex cases with greater variations, but this is not the case here. Note that, despite the increase in the number of networks, the calculation time to obtain the state of the tool is less than a second, which enables real-time monitoring.

## 4. Conclusions

In this paper, a bootstrap-based neural network approach for monitoring the degradation of turning tools is proposed. The approach is able to accurately track and detect the end of life of tools in a timely manner with an estimation of the confidence interval on this prediction. The wear criterion used to monitor tool degradation is the VB value, as defined in ISO 3685.

The database used to test this approach contains cutting force signals acquired over the degradation of 30 tools with different cutting speeds. The five features used as inputs to the network are $M_{Z}$ RMS, $F_{X}$ RMS, machining duration, total machined length, and $F_{Z}$ RMS. These inputs all have a correlation close to 80% with tool wear, which makes them good wear indicators.

The identification of the best network architecture and hyperparameters is carried out by comparing the trends in the literature and adjusting these trends to the presented dataset. With the database presented in this paper, it appears that the best architecture contains, in its first hidden layer, six neurons with the activation function “Tanh”; there is also a second hidden layer comprising six neurons but with the activation function “ReLu”. The network is therefore composed of five inputs followed by the two hidden layers and an output layer composed of a single neuron representing the indicator VB.

The main novelty of the approach is that is takes into account the confidence interval around the prediction with a bootstrap method. This approach consists of training several neural networks in parallel and considering the results of these networks as a normal distribution. The normal distribution hypothesis is demonstrated, and this enables the calculation of a confidence interval. It is particularly useful as it can help in the decision for optimal tool replacement policies.

The bootstrap approach significantly increases the computational resources needed to train neural networks; however, with the current computational and parallelization capabilities and the limited size of the network, the impact on training time is not very high. In the presented approach, the parallelization of the training enables the learning to be up to six times faster; this brings the computation time below 15 min. Once the networks are trained, the prediction takes less than a second to complete. As the learning only needs to be performed once, this validates the approach for online wear estimation. To obtain the results, 100 networks were trained; this number was chosen in an effort to ensure that there was a sufficient statistical distribution—this has not been optimized. It is therefore possible to greatly reduce the training time by comparing the performances with a different number of networks.

The approach was tested on degradation trajectories with variable cutting speeds. The obtained results show an excellent correlation between the estimated tool degradation and the real value measured on the tool. On average, the error between the prediction and the measured value is a few microns; this has no impact on the machining process as such. It is also observed that the evolution of the confidence interval is the direct image of the certainty of the network in its prediction. The size of this interval evolves coherently with the situation. Indeed, when the network deviates from reality, the size of the interval increases; this signifies an increase in the uncertainty of the network. In the majority of cases, the confidence interval is small (a few microns); this is a promising result for future work. Indeed, the database used in this article has little variation in cutting condition. Having a larger confidence interval would have been a sign that this method could not be used in more complex cases. The trends also show that the approach tends to give conservative results by slightly overestimating the wear in the majority of the cases, which is safer for industrial applications.

Future work should focus on generalizing this approach to larger variations in cutting conditions than those presented in this paper. In addition, limiting the number of networks used in parallel would reduce the computing resources needed to train these networks.

## Figures and Tables

**Table 1:** Cutting conditions for each turning test.

Test N°	Material	Cutting Speed—$V_{c}$ [m/min]	Feed [mm/rev]	Depth of Cut [mm]
1 to 10	C45	260	0.2	1
11 to 15	C45	250	0.2	1
16	C45	240	0.2	1
17 to 20	C45	265	0.2	1
21 to 30	C45	Variable: 240 to 260	0.2	1

**Table 2:** Experimental parameters for each turning test.

Parameters Measured	Associated Variables
Cutting force and Torque	$F_{x}$, $F_{y}$, $F_{z}$, $M_{x}$, $M_{y}$ and $M_{z}$
Time	Machining time
Picture of the tool	VB and notch wear

**Table 3:** Statistical features processing.

Feature Processing Method	Mathematical Equation
Mean ($\bar{x}$)	$m_{1}$
Standard deviation (σ)	$\sqrt{m_{2}}$
Skewness (skew)	$\frac{m_{3}}{m_{2}^{3/2}}$
Kurtosis (kurt)	$\frac{m_{4}}{\sigma^{4}}$
Root Mean Square (RMS)	$\sqrt{\frac{1}{N}\sum_{i=1}^{t}t_{i}^{2}}$

**Table 4:** Most correlated features with respect to the tool wear.

Features	Spearman’s Correlation Coefficient with Respect to VB
$M_{z}RMS$	0.89
$F_{x}RMS$	0.87
Machining duration	0.84
total machined length	0.84
$F_{z}RMS$	0.79

**Table 5:** Software used to create the neural network.

Version	Task
Python 3.8.11	Programming language
Keras 2.4.3	NN API
Tensorflow 2.3.0	Open source platform for machine learning
Matplotlib 3.4.2	Visualization
Pandas 1.2.5	Data manipulation
Scipy 1.6.2	Mathematical computation
Numpy 1.22.3	Array manipulation

**Table 6:** Comparison of the relative performance of some architectures after maximum 1000 epochs.

Network Architecture	MSE	R^2^ Score	MAPE	Activation Function
[5, 6, 6, 1]	850	94.5%	15%	Tanh and ReLu
[5, 6, 10, 6, 1]	1200	91%	19%	Tanh, ReLu and ReLu
[5, 6, 6, 1]	1250	90%	25%	Sigmoid and ReLu
[5, 5, 13, 9, 1]	1330	90%	23%	All ReLu
[5, 11, 1]	1550	89%	22%	All ReLu
[5, 6, 1]	1600	88%	27%	All ReLu
[5, 12, 5, 1]	1630	88%	25%	All ReLu
[5, 6, 6, 1]	1700	87%	26%	All ReLu
[5, 6, 6, 1]	12,000	14%	30%	All Tanh
[5, 6, 6, 1]	12,000	14%	30%	All Sigmoid

## Data Availability

The datasets presented in this article are not readily available because the data are part of an ongoing study. Requests to access the datasets should be directed to the corresponding author.
